# Developing a Workflow to Identify Inconsistencies in Volunteered Geographic Information: A Phenological Case Study

**DOI:** 10.1371/journal.pone.0140811

**Published:** 2015-10-20

**Authors:** Hamed Mehdipoor, Raul Zurita-Milla, Alyssa Rosemartin, Katharine L. Gerst, Jake F. Weltzin

**Affiliations:** 1 Faculty of GeoInformation Science and Earth Observation (ITC), University of Twente, Enschede, The Netherlands; 2 School of Natural Resources and the Environment, College of Agriculture and Life Sciences, University of Arizona, Tucson, Arizona, United States of America; 3 USA National Phenology Network, National Coordinating Office, Tucson, Arizona, United States of America; 4 United States Geological Survey, Tucson, Arizona, United States of America; Peking UIniversity, CHINA

## Abstract

Recent improvements in online information communication and mobile location-aware technologies have led to the production of large volumes of volunteered geographic information. Widespread, large-scale efforts by volunteers to collect data can inform and drive scientific advances in diverse fields, including ecology and climatology. Traditional workflows to check the quality of such volunteered information can be costly and time consuming as they heavily rely on human interventions. However, identifying factors that can influence data quality, such as inconsistency, is crucial when these data are used in modeling and decision-making frameworks. Recently developed workflows use simple statistical approaches that assume that the majority of the information is consistent. However, this assumption is not generalizable, and ignores underlying geographic and environmental contextual variability that may explain apparent inconsistencies. Here we describe an automated workflow to check inconsistency based on the availability of contextual environmental information for sampling locations. The workflow consists of three steps: (1) dimensionality reduction to facilitate further analysis and interpretation of results, (2) model-based clustering to group observations according to their contextual conditions, and (3) identification of inconsistent observations within each cluster. The workflow was applied to volunteered observations of flowering in common and cloned lilac plants (*Syringa vulgaris* and *Syringa x chinensis*) in the United States for the period 1980 to 2013. About 97% of the observations for both common and cloned lilacs were flagged as consistent, indicating that volunteers provided reliable information for this case study. Relative to the original dataset, the exclusion of inconsistent observations changed the apparent rate of change in lilac bloom dates by two days per decade, indicating the importance of inconsistency checking as a key step in data quality assessment for volunteered geographic information. Initiatives that leverage volunteered geographic information can adapt this workflow to improve the quality of their datasets and the robustness of their scientific analyses.

## Introduction

The contribution of volunteers to the production of information about geographic phenomena, such as the impacts of climate change, is not new. For example, the Christmas Bird Count has studied the impacts of climate change on spatial distribution and population trends of selected bird species in North America since 1900 [1]. However, improvements in online information communication and mobile location-aware technologies have led to a dramatic increase in the amount of volunteered geographic information (VGI) in recent years [2–5]. VGI, a term coined by Goodchild [2], refers to "the harnessing of tools to create, assemble, and disseminate geographic data provided voluntarily by individuals". VGI is a practical approach to acquire timely and detailed geographic information at low cost across a variety of spatial and temporal scales [6]. Because of this, VGI is used to understand and manage important emerging problems in many fields such as conservation biology [7], urban planning [8], disaster management [9] and earth observation [10–12].

Despite the wide applicability and acceptability of VGI in science [4, 13], many studies argue that the quality of the observations provided by volunteers remains a concern [6, 14–21]. This is because VGI does not often follow scientific principles of sampling design, and levels of expertise vary among volunteers [22, 23]. Moreover, unlike traditional authoritative geographic information, VGI typically lacks automated quality checking mechanisms [24–28]. Among the different data quality aspects, consistency of VGI is considered key for most studies, where inconsistent VGI are observations that are implausible regarding the conditions, geographic location or time they were obtained. Such inconsistent observations can bias analysis and modeling results because they are not representative for the variable studied, or because they decrease the ratio of signal to noise. Hence, the identification of inconsistent observations would clearly benefit VGI-based applications and provide more robust datasets to the scientific community.

The approaches to check VGI quality can be categorized into three main types [6, 20]: 1) crowdsourcing where volunteers validate and thus refine the quality of observations by themselves, 2) social which relies on a hierarchy of trusted people who act as moderators, and 3) geographic, where given the location of the volunteered observations, one can use certain geographic rules to assess quality, e.g., Tobler's “first law of geography” which states that “all things are related, but nearby things are more related than distant things” (Tobler, 1970). The geographic approach is more readily machine-automated than the other two approaches (which rely on human subjectivity) [6], and is therefore the focus of this study.

As an example, eBird, a popular VGI-based initiative for bird monitoring, uses the geographic approach to automatically verify new observations, using historical observations, prior to human moderation [29]. The eBird quality filter relies on substantial prior knowledge about a given organism, geography or time (e.g., a measure of how frequently a species is reported in a region during a specific time period), as well as information about volunteer expertise levels [25]. Such information is not always available for VGI-based initiatives.

Schlieder and Yanenko [30] used spatiotemporal proximity and social distance (i.e. the distance between the observers in the social network of observers on the web) to define constraints for checking the inconsistency of observations. The hypothesis was that spatiotemporally and socially close observations presumably referred to the same event so would more likely be consistent. Their workflow was used to formulate general rules and to find observations that have low confirmation. This workflow was further developed using constraint satisfaction approach to produce more sophisticated results [31]. However, the improved workflow still uses spatial distance as the only criterion to connect observations. Moreover, this workflow is useful only when sequential order of volunteered observations is available at a given location.

Yet another geographic workflow was proposed by Ali and Schmid [32] based on machine learning for identifying wrongly-categorized Open Street Map observations. These authors trained a classifier using contributed entities and their associated class labels (e.g., park or garden). However, their model was only concerned with the inconsistency of areal entities (i.e., extended geometric entities such as buildings) regarding administrative boundaries and semantic classifications.

There is a lack of standardized workflows that address VGI inconsistency. Current inconsistency workflows primarily rely on human review, or simple statistical deviation from an expected probability distribution. Human-dependent workflows can be costly and time-consuming, and are impracticable in some situations, e.g., in cases where events persist only for short periods of time. The statistical workflows assume that the majority of the observations are consistent and, therefore, that these can be used to check for inconsistency. Moreover existing workflows do not optimally use environmental contextual data. This raises the question of how to address inconsistency using a more objective, efficient and automated workflow.

This paper describes a novel automated workflow to identify inconsistency in VGI. A robust identification of inconsistent observations allows testing their potential impact on VGI-based studies. The workflow relies on the availability of contextual information and is built using a combination of dimensionality reduction, clustering and outlier detection techniques and it was illustrated using observations on the timing of the first flower of lilac plants collected by volunteers. While some inconsistent observations may reflect real, unusual events, here we demonstrate that these observations bias the trends (advancement rates) of the date of lilac flowering onset. This shows that identifying inconsistent observations is a pre-requisite to study and interpret the impact of climate change on the timing of life cycle events [33, 34].

## Materials and Methods

### Phenological VGI

Phenology is the science of the study of periodic plant and animal life cycle events and how seasonal and inter‐annual variations in climate affect them. Phenological studies are important to understand the impact of global change in our planet [35–38]. Worldwide, several VGI-based initiatives collect or have collected phenological data [39, 40]. One VGI-based initiative, the USA National Phenology Network (USA-NPN; www.usanpn.org), has recently released a curated dataset of lilac leafing and flowering observations across the continental United States for the period 1956 to 2014 [41]. From this dataset we extracted flowering records for common lilac (*Syringa vulgaris*) and cloned lilac (*S*. *x chinensis ‘*Red Rothomagensis’). Considering data completeness and the availability of environmental contextual data, we concentrated our analyses on flowering onset dates for the period 1980 to 2013, for cloned lilacs (with 2174 observations) and common lilacs (with 2682 observations) separately.

Widespread and readily observable, lilac plants have been observed across the continental United States since the 1950’s, as a complement to cooperative weather data collection [42]. Observations of lilac leafing, flowering and fruiting have been used for a variety of applications, including understanding trends and variations in the onset of spring and tracking the impacts of climate change on natural resources[43]. Although lilacs are ornamental plants, their phenology and response to climate have been shown to closely track native species and crops [33].

The following attributes were used to check inconsistency for cloned and common lilac flowering dates: (1) a unique ID for each record, (2) the year when the flowering occurred, (3) the day of the year (DOY) when the flowering occurred and (4) geographic location where the phenological phase was reported (latitude, longitude and elevation). It is important to note that since 2009, volunteers report the status of each phenological phase with”Yes” when it is visible and “No” when it is not visible [44]. This status monitoring approach allows for the quantification of uncertainty in flowering onset DOYs (i.e., number of days between the “Yes” and the preceding “No”). Thus, the status monitoring provides additional information on the occurrence of multiple flowering events in a year for individual plants. When a “Yes” report was followed by at least one “No” report and then a subsequent “Yes” record was present on an individual plant, all corresponding DOYs to “Yes” reports were flagged and stored as multiple “Yes” observations in the dataset.

### Environmental contextual data

The proposed workflow requires environmental contextual data to characterize observation locations. In phenology, cumulative climatic parameters are the most relevant contextual datasets, because most phenological processes are driven by climate conditions [37, 45, 46]. Therefore, we extracted climate parameters for the period 1980 to 2013 from DAYMET, a dataset that provides 1 km by 1 km gridded estimates of daily climatic parameters for North America [47].

Cumulative climatic variables were created for each geographic location by summing parameter values from the 1 January for the year of the observation to the reported DOY of flowering. Cumulative variables calculated include: maximum daily temperature (degrees C), minimum daily temperature (degrees C), daily precipitation (mm/day), daily water vapor pressure (Pa), daily solar radiation (W/m^2^), daily day length (s/day) and daily snow water equivalent (kg/m^2^). In addition, using the daily maximum and minimum temperatures, we calculated daily average temperatures and cumulative average daily temperature (degrees C). Thus, a total of 11 contextual variables (i.e., 8 cumulative climatic variables and the 3 geographic variables of latitude, longitude and elevation) were associated with each phenological observation expressed as DOY (Table 1).

**Table 1:** Mean and standard deviation of the geographic and climatic parameters for cloned and common lilacs.

Variable	Cloned lilac		Common lilac	
	Mean	Standard deviation	Mean	Standard deviation
**DOY (of flowering)**	133	21	123	22
**Latitude**	42.42	2.46	42.13	4.15
**Longitude**	-79.38	9.73	-105.60	12.99
**Elevation**	255.86	252.79	917.51	1051.14
**Cumulative maximum daily temperature**	934.59	535.02	1058.95	406.49
**Cumulative minimum daily temperature**	-547.59	465.42	-522.19	469.43
**Cumulative average daily temperature**	542.89	372.66	506.69	207.62
**Cumulative daily day length**	5512631	1044335	5000749	1083965
**Cumulative daily precipitation**	348.47	151.50	222.39	205.54
**Cumulative daily solar radiation**	43261.2	8504.76	40775.14	11737.74
**Cumulative daily snow water equivalent**	5321.42	6187.48	3106.81	7462.61
**Cumulative daily water vapor pressure**	69509.64	31508.84	52258.41	19946.21

### The context-aware workflow

The proposed context-aware inconsistency check workflow builds upon elements from existing workflows. More precisely, it relies on the wide availability of contextual (environmental and geographic) information, enabling us to characterize complex differences between observation locations in space and time. When this characterization results in a high-dimensional dataset, the data are mapped to a low-dimensional space to facilitate the subsequent analysis of the data and the visualization of the results. Next, observations are clustered into contextually homogenous subsets. Finally, inconsistent observations are identified by analyzing the outliers present in each cluster.

#### Dimensionality reduction

The t-distributed stochastic neighbor embedding (t-SNE) algorithm [48] was selected to reduce the dimensionality of the contextual information. This algorithm maps the data to a low-dimensional space, typically two or three dimensions, so that data visualization is possible. It retains the local structure of the data which means that similar objects are mapped to nearby points in the low-dimensional space. Moreover, the model-based clustering step of the workflow has limited ability to deal with high-dimensional data, which further justify the use of the t-SNE algorithm.

The t-SNE defines a probability distribution over pairs of data points in the high-dimensional space so that similar ones have a high probability of being selected. Next, the t-SNE defines a similar distribution over the data points in the low-dimensional space in such a way it minimizes the information lost when such distribution is used to approximate the distribution in high-dimensional space. In particular, t-SNE uses the Kullback–Leibler divergence [49] which quantifies the difference between the two probability distributions (in this case, those of the original and of the low dimensional data points).

The t-SNE algorithm requires the definition of the perplexity value, which is a smooth measure of the effective number of neighbors used to define the probability distribution in the high- and low-dimensional spaces. However, typical perplexity values are located in a limited interval (between 5 and 50) so optimizing its value is relatively easy. We used the “t-SNE” R package to perform all calculations in this study [50].

#### Model-based clustering

Model-based clustering [51, 52] was selected to cluster the contextual information because it automatically identifies the number, shape and size of the clusters present in a dataset. This increases the objectivity of the analysis by reducing the need for human intervention and facilitates its use for multiple applications. The automated identification of cluster characteristics is realized by sequentially fitting several mixture models [53] to the dataset and selecting the one that maximizes the Bayesian Information Criterion (BIC) [54]. We calculated the BIC values for ten Gaussian mixture models currently available in the R package, “mclust” [55].

The uncertainty of the clustering was calculated (by subtracting the probability of the most likely group for each data point from one) and analyzed to determine its impact on the identification of inconsistent observations. Data points with an uncertainty value of more than 0.5 were ignored as they could be either an inconsistent or a mis-clustered observation.

The model-based clustering method implemented in “mclust” uses the Expectation Maximization (EM) algorithm [56]. The EM, an iterative method, is used to find maximum likelihood parameters of a mixture model, specifying the mixture component to which each data point belongs. This algorithm is relatively robust but its efficiency is negatively affected by the dimensionality of the input data because the number of parameters that need to be estimated is proportional to the dimensionality of the data [55].

#### Intra-cluster outlier detection

**Fig 1:**
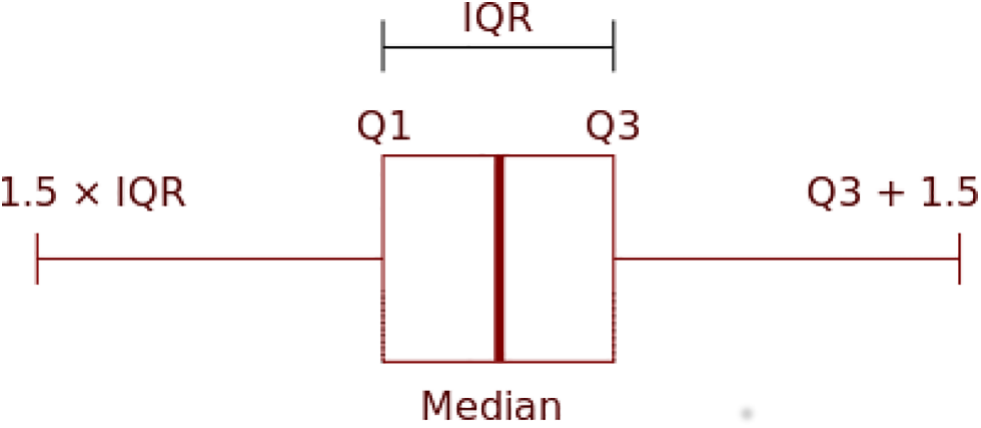
The Tukey boxplot.

The identification of inconsistent observations requires defining objective and easily automatable rules. Here we used the Tukey boxplot as a main tool to highlight inconsistent observations [57]. The boxplot is a hybrid non-parametric method that displays variation and outliers in numerical data by visually indicating its degree of dispersion and skewness in the data (Fig 1). The bottom and top of the box represent the first (Q_1_) and third (Q_3_) quartiles of the data respectively, and the band inside the box represents the second quartile (the median).

In the Tukey boxplot the whiskers cover 150% of the interquartile range (i.e. 1.5 x IQR). If the numerical data are normally distributed, points larger or smaller than the values represented by the whiskers are 0.7% of the data and are typically considered outliers [57]. In this study, these outliers are highlighted as inconsistent observations. The outlier detection is also done using the built-in function of boxplot in the R software package to create an automated and clean workflow that can be re-used for multiple applications.

#### Impact of inconsistent observations

To investigate the impact of the inclusion of inconsistent observations in an analysis of phenological patterns, we used linear regression to model the trend in the flowering onset DOY–with and without inconsistent observations–over the complete study period. Regression models were developed for pooled observations of cloned and common lilacs, and separately for each type of lilac. Finally, we used analysis of covariance [58] to test the effect of the inconsistency of observations (i.e., consistent and inconsistent) on flowering onset DOY while controlling for the effect of the year of observations. This analysis is used to statistically test for differences in slopes among regression models. The regression modeling and the covariance analysis were done using built-in functions of the R software package.

## Results and Discussion

**Fig 2:**
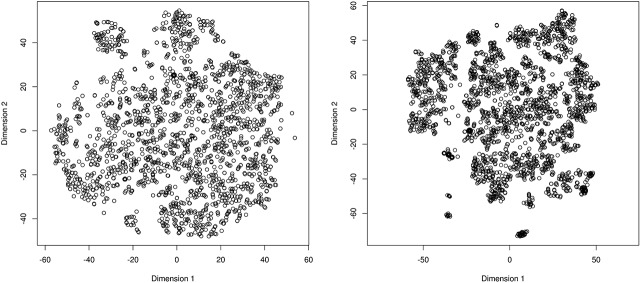
The results of applying t-SNE on contextual information. The transformed contextual information for (A) cloned lilac and (B) common lilac.

The eleven-dimensional data space that characterizes the phenological observation was transformed to a two-dimensional space (Fig 2) while testing several perplexity values (5 to 50 in steps of 5 units). The optimal perplexity value was chosen as the one that maximizes clustering (i.e. the one that better “spreads” and “separates” the observations into distinct groups). For both datasets, the perplexity value equaled 35, which led to the maximum number of clusters that the EM algorithm could identify.

A visual inspection of the transformed data space in Fig 2 shows that the environmental conditions of the observation sites for cloned lilac are similar to each other, as the majority of points formed a cloud shape. It also shows that the observation sites for the common lilac are more clustered, indicating that these observations are made in more contrasting environments [59] relative to the cloned lilacs [60]. This is consistent with the fact that cloned lilacs were only observed in the Eastern U.S. [57], which is characterized by less environmental variability than the Western U.S. (Table 1).

**Fig 3:**
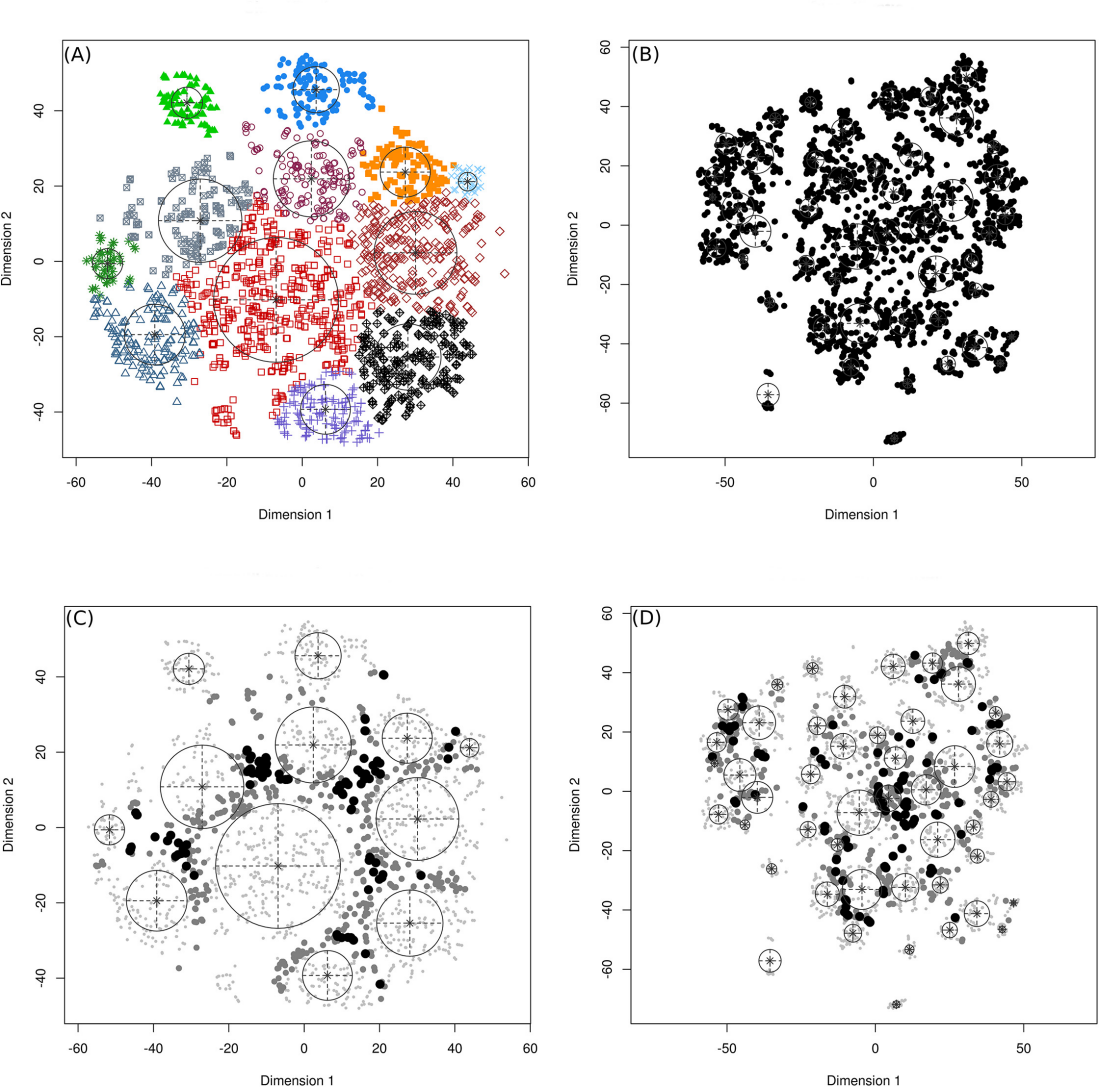
The results and uncertainty of model-based clustering. Clusters of the transformed contextual information about (A) cloned lilac and (B) common lilac. The uncertainty in clustering of transformed contextual information about (C) cloned lilac and (D) common lilac. In uncertainty plot, the symbols have the following meaning: large filled symbols, 95% quantile of uncertainty; smaller open symbols, 75–95% quantile; small dots, first three quartiles of uncertainty.

As expected from the t-SNE results, the number of clusters for the common lilac (47 clusters) is larger than for the cloned lilac (12 clusters). These results (Fig 3) demonstrate that a diagonal Gaussian mixture distribution—-with equal shape, variable volume and coordinate axes orientation—-fits best the contextual information for both cloned and common lilacs (Table 2).

**Table 2:** The fitted mixture models currently in the “mclust” package and their corresponding BIC values.

Distribution	Volume	Shape	Orientation	BIC of cloned lilac	BIC of common lilac
Spherical	Equal	Equal	-	-40096	-49152
Spherical	Variable	Equal	-	-39977	-48801
Diagonal	Equal	Equal	Coordinate axes	-40130	-49124
Diagonal	Variable	Equal	Coordinate axes	**-39905**	**-48768**
Diagonal	Equal	Variable	Coordinate axes	-40091	-49307
Diagonal	Variable	Variable	Coordinate axes	-40082	-48950
Ellipsoidal	Equal	Equal	Equal	-40119	-49172
Ellipsoidal	Equal	Equal	Variable	-40115	-49203
Ellipsoidal	Variable	Equal	Variable	-40130	-48960
Ellipsoidal	Variable	Variable	Variable	-40036	-49268

**Fig 4:**
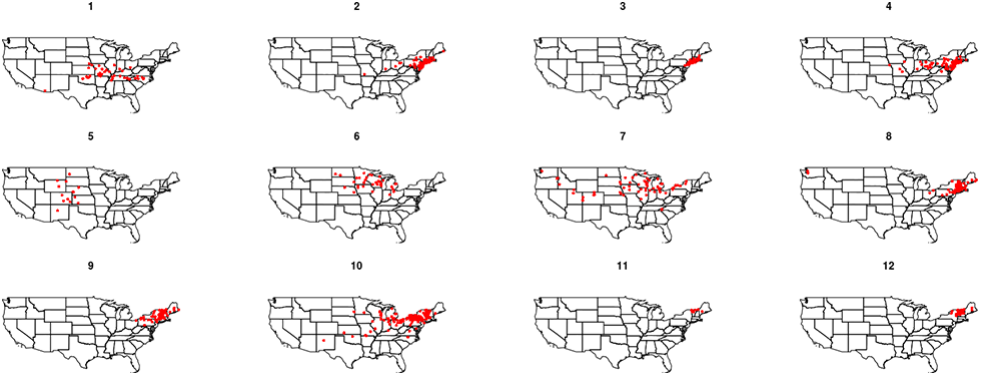
The geographic distribution of the clusters in context condition of cloned lilac.

**Fig 5:**
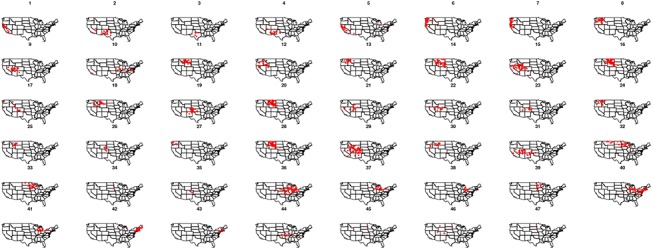
The geographic distribution of the clusters in context condition of common lilac.

The phenological observations belonging to each cluster were projected into the geographic space to study their geographic distribution (Figs 4 and 5). For both types of lilac, the observation sites that belong to the same cluster are often spatially clustered (i.e., clusters tend to be compact). Nevertheless, there are some sparse clusters (e.g., cluster 7 and 10 of cloned and clusters 29,31, 32, 36 and 40 of common lilac) that indicate geographically distant observation sites with similar climatic context.

**Fig 6:**
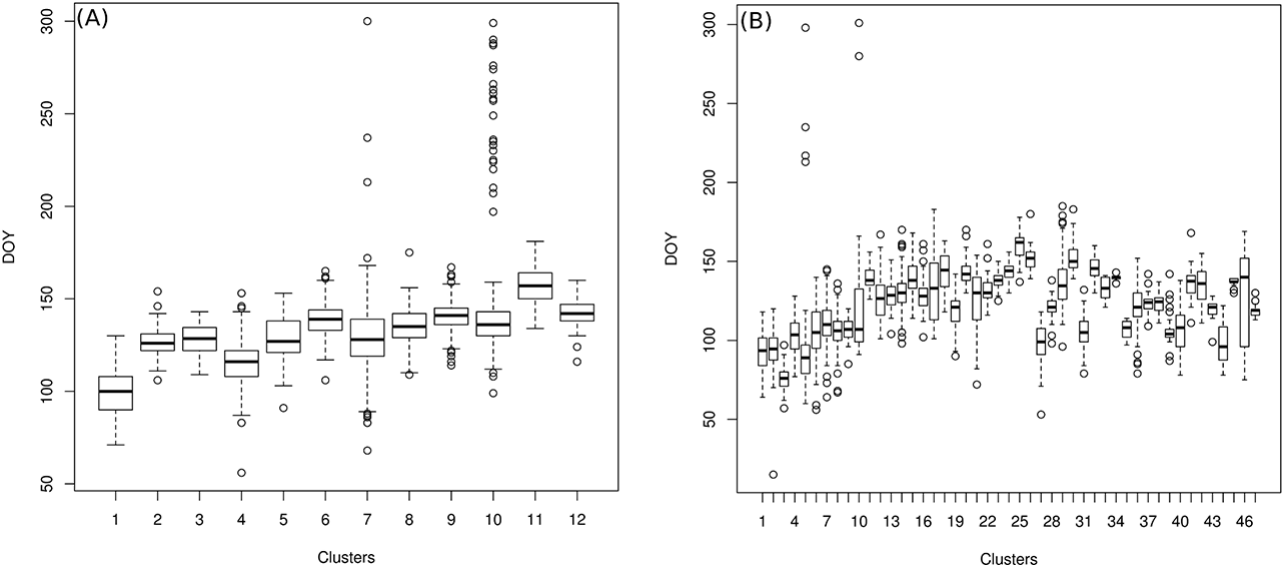
Intra-cluster boxplot of DOYs that lilac started flowering. Boxplots of corresponding DOYs in clusters of transformed contextual information for (A) cloned lilac and (B) common lilac. Hollow circles represent intra-cluster outliers.

The variability across the interquartile ranges and median values of the clusters for common lilacs is greater than for cloned lilac (Fig 6). The greater variability in observations on common lilac reported from the Western U.S. was expected based on the clusters described above, and has been noted in other studies [22, 61]. The outliers identified by the boxplots were highlighted as inconsistent phenological observations in this study.

**Fig 7:**
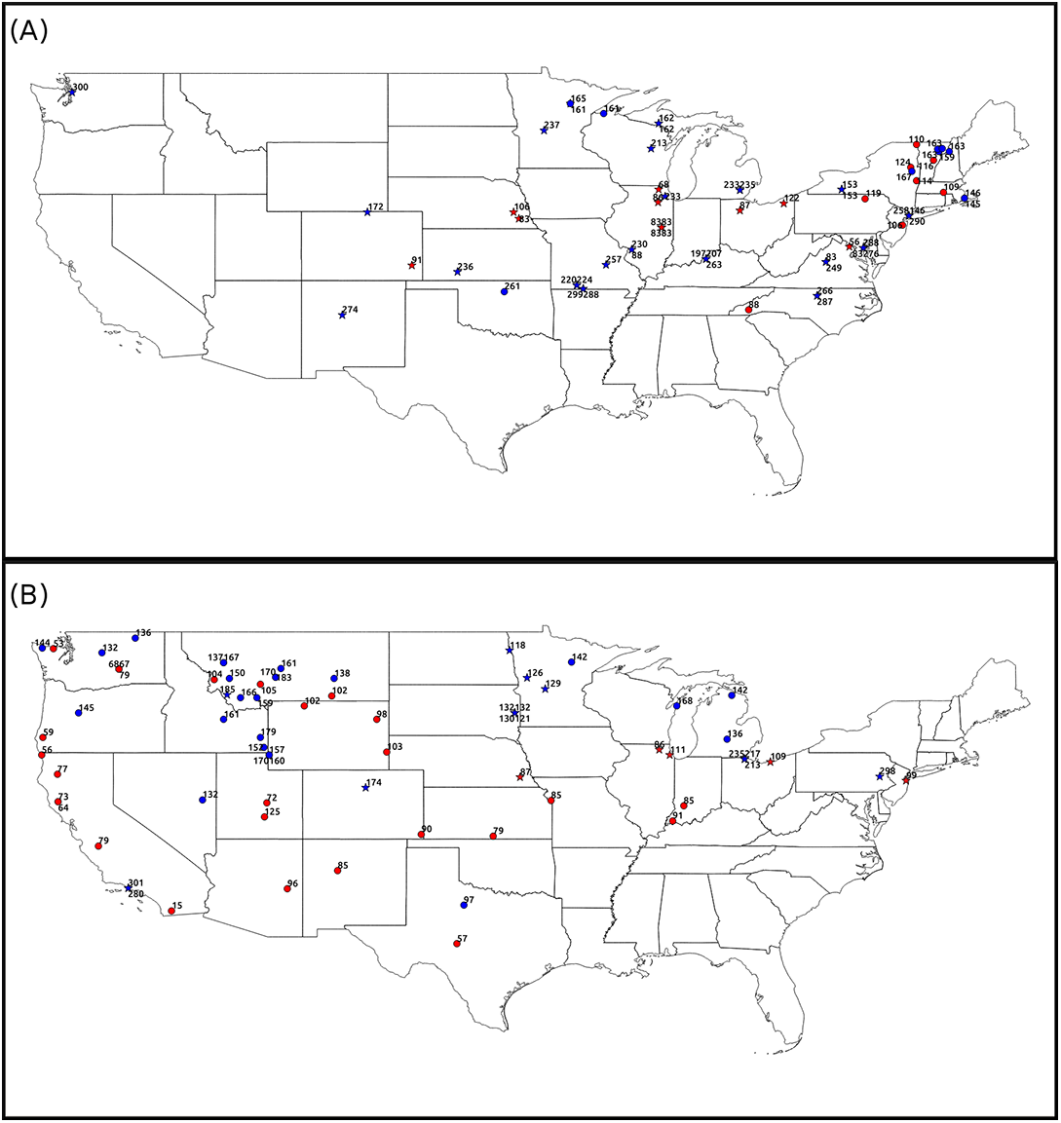
Plot of inconsistent phenological observations through study area. Inconsistent volunteered observations on flowering onset DOY of (A) cloned lilac and (B) common lilac. Red points show unusually early while blue ones show unusually late phenological observation. Circles show that phenological observations from historical initiatives whereas stars show phenological observations from contemporary initiatives. Inconsistencies were labeled with the day of year that lilac started flowering.

Inconsistent observations were found in both pre- and post-2009 phenological observations (Fig 7). For both types of lilacs, the highlighted inconsistencies accounted for about 3% of phenological observations (3.1% and 2.9% of phenological observations on cloned and common lilac respectively). 53% of the inconsistent observations on cloned lilacs have greater than one week uncertainty (>7 days between the prior “No” and the first “Yes” observation) whereas less than 15% of inconsistent observation on common lilac have greater than one week uncertainty in the estimated onset DOYs. Moreover, 41% of the inconsistent observations of cloned lilac and 50% of the common lilacs are associated with sites that report multiple flowering in a year (post 2009, when reports of repeat flowering were allowed, e.g., to account for flowering activity after frosts).

The unusually late “Yes” observation are not necessarily a result of erroneous data collection, because lilacs can also flower in the autumn (which may be associated with different environmental factors). In addition, unusually early “Yes” reports preceded by a second consistent “Yes” spring record might point to mild winter in which lilacs start flowering early, experience frost, and then set flower again. For example, in 2012 in Charlottesville, Virginia, first flowering of a cloned lilac shrub was reported in February (i.e., early relative to other observations at the site). The flowering of the shrub was also reported later, on April 7^th^, which is more consistent, as determined by the workflow.

**Fig 8:**
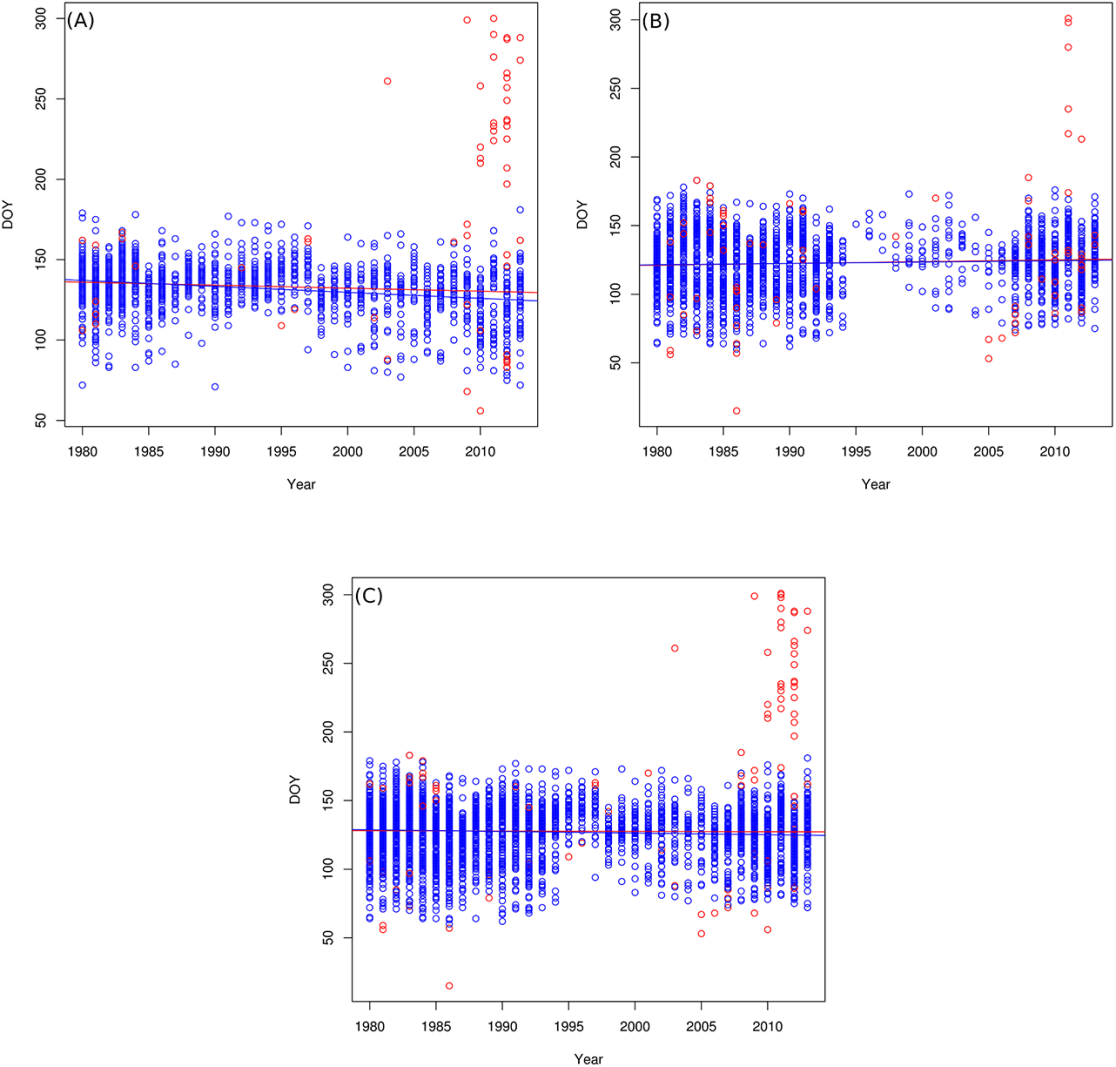
Comparison of the linear modeling of the original phenological observations and the consistent phenological observations. Temporal trends in the flowering onset DOY of (A) cloned lilac, (B) common lilac, and (C) pooled observations of cloned and common lilac.

For cloned lilacs, the rate of change in flowering onset DOY (i.e., the slope of the regressions) significantly (P < 0.001) changed from -0.19 to -0.37 when inconsistent observations were excluded. In other words, using the cleaned dataset for the trend analysis resulted in two days additional advancement per decade in flowering onset of cloned lilac compared to the raw dataset. Likewise, for common lilacs, excluding inconsistent observations affected the regression slope, but to a lesser degree (from 0.12 to 0.9; P = 0.06) than in the cloned lilacs. For the pooled observations, the slope changed from -0.02 to -0.12 (P < 0.001) when the inconsistent observations were removed, resulting in one additional day advancement per decade in flowering onset across the U.S.. Thus, the inclusion of inconsistent observation underestimates the rate of acceleration of the lilac onset dates over the period 1980–2013 (Fig 8). These results are in agreement with previous studies that found a gradual advance in the flowering onset DOYs [22, 34].

## Conclusions

The identification of inconsistent observations is a pre-requisite for any kind of analysis or modeling effort. In this paper, using a phenology case study, we present and demonstrate a computational workflow that has potential to automate the identification of inconsistencies in data collected by VGI-based initiatives. The workflow relies on environmental data as critical context that affects the variability in the observational datasets, and consists of a sequence of dimensionality reduction, model-based clustering and outlier detection.

The workflow demonstrated that we can highlight unusually early or late observations of the flowering onset DOYs for lilacs. The identified inconsistencies should be further analyzed using more granular climate data or expert knowledge to determine if they are likely observation or transcription errors or represent truly anomalous events, due to microclimate, or genetic variation, in the case common lilacs. Overall low inconsistency rate (about 3%) indicates that volunteer collected observations are a valuable source of information for the study of phenology.

Phenological VGI has greatly contributed to our understanding of seasonal spatial and temporal patterns for plants and animals across the globe. Given that phenology has been recognized as an important indicator of climate change and has emerged as a vibrant area of research at multiple ecological scales, analyses that increase data quality and usability will greatly benefit the fields of climate research, ecology, and natural resource management. We envision that this workflow will greatly increase the reliability of, and potential for scientific contribution from, spatially and temporally rich VGI datasets.

Focusing subsequent analysis on the inconsistent observations identified by our workflow reduces human checks, which saves money and time. Moreover, unlike existing workflows, the proposed workflow uses relevant contextual information for the phenomena under study (as climate drives phenological events). Therefore, we recommend that initiatives collecting volunteered geographic information use the proposed automated workflow and relevant contextual information to check inconsistency in order to improve data quality. This workflow could be applied to volunteered meteorological data [62] to, for instance, highlight unusually high or low temperature reports because daily weather data has a long history and is increasingly available [63].
